# Microbial Adherence to Contact Lenses and *Pseudomonas aeruginosa* as a Model Organism for Microbial Keratitis

**DOI:** 10.3390/pathogens11111383

**Published:** 2022-11-19

**Authors:** Allison Campolo, Reed Pifer, Paul Shannon, Monica Crary

**Affiliations:** Alcon Research, LLC, Fort Worth, TX 76134, USA

**Keywords:** contact lenses, microbial adherence, *Pseudomonas aeruginosa*

## Abstract

Microbial keratitis (MK), the infection of the cornea, is a devastating disease and the fifth leading cause of blindness and visual impairment around the world. The overwhelming majority of MK cases are linked to contact lens wear combined with factors which promote infection such as corneal abrasion, an immunocompromised state, improper contact lens use, or failing to routinely disinfect lenses after wear. Contact lens-related MK involves the adherence of microorganisms to the contact lens. Therefore, this review discusses the information currently available regarding the disease pathophysiology, the common types of microorganisms causing MK, physical and organic mechanisms of adhesion, material properties which are involved in adhesion, and current antimicrobial strategies. This review also concludes that *Pseudomonas aeruginosa* is a model organism for the investigation of contact lens microbial adherence due to its prevalence in MK cases, its extremely robust adhesion, antimicrobial-resistant properties, and the severity of the disease it causes.

## 1. Introduction

Microbial keratitis (MK) is a serious, sight-threatening disease, and its severity is often dictated by accumulation of biofilms on external contact lenses [1]. It is estimated that two-thirds of human infections involve biofilms, including infections involving the urinary tract, lungs, ears, dental plaque, implants, and contact lenses [2]. Biofilms are a major concern in the field of medical devices in general, and both gram-positive and gram-negative bacteria can form biofilms on medical devices [3]. The most common microorganism species that form these biofilms on medical devices include *Pseudomonas*, *Enterococcus*, *Staphylococcus*, *Streptococcus*, *Klebsiella*, and *Serratia* [4]. Critically, it has been shown that implantable devices are relatively easy to contaminate due to the fact that they require roughly 10,000 times less bacterial load to colonize a medical device compared to host tissue [5]. Thus, designing devices that are easily disinfected before use or that microorganisms have difficulty colonizing is key.

## 2. Microbial Keratitis

Microbial keratitis (MK) is a serious ocular infection which afflicts the cornea and can potentially lead to blindness. To note, the term “microbial keratitis” encompasses bacterial, fungal, and amoeba keratitis. The most prominent risk factor for MK is contact lens wear, resulting from patients who do not properly clean their lenses or from ineffective contact lens care solutions [6]. Other risk factors for MK include ocular surface disease, ocular trauma, and ocular surgery [7]. Regardless of cause, *Pseudomonas aeruginosa* is the most commonly isolated gram-negative organism from MK cases, particularly in contact lens-associated MK [8,9]. This is largely attributed to *P. aeruginosa* being such a ubiquitous pathogen and also to it being such a versatile one. *P. aeruginosa*’s versatility stems from a complex regulatory network and a wide range of virulence factors, which include motility and the secretion of proteases and toxins [8]. This pathogen is also particularly challenging due to the high incidence rate of antibiotic-resistant strains [8]. 

**Figure 1:**
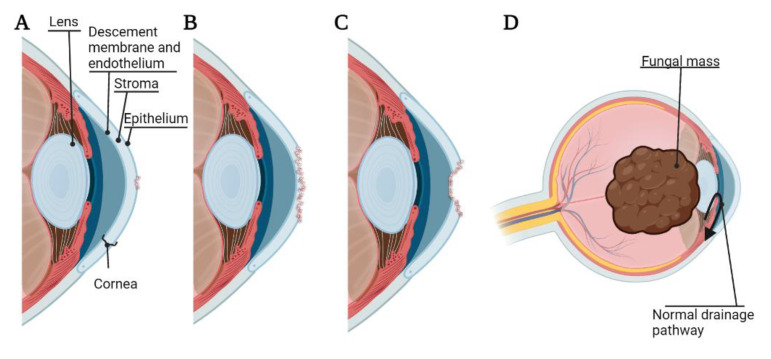
Pathogenesis and adverse events of microbial keratitis. (**A**) Corneal ulcer, damage to the corneal epithelium; (**B**) Corneal melting, destruction of the corneal stroma; (**C**) Corneal perforation, damage extended through the corneal endothelium; (**D**) Malignant glaucoma caused by fungal mass pressing on the lens and occluding natural drainage of aqueous humor. The figure is created using BioRender.com. (accessed on 18 March 2022).

Pathogenesis of bacterial, fungal, or *Acanthamoeba* keratitis begins with adhesion of the microorganism to the cornea. The binding of these organisms to the cornea can lead to desquamation of the corneal epithelium, and the cytopathic effect can proceed via cytolysis, phagocytosis, and apoptosis [10]. Microorganisms can also secrete proteases and exotoxins, resulting in the degradation of the basement membrane and extracellular matrix by lysing cells [11]. In general, once a microorganism has sufficiently adhered to the corneal surface under vulnerable conditions (such as corneal abrasion and/or contaminated contact lenses), this can lead to corneal ulcer, corneal melting, and corneal perforation [11]. Further, while bacterial and *Acanthamoeba* keratitis rarely invade the eye beyond the corneal epithelium [12], fungal keratitis is able to gain access to the anterior chamber of the eye leading to a lens-iris-fungal mass affecting drainage of the aqueous humor and leading to fungal malignant glaucoma [13] (Figure 1). Interestingly, the innate ocular microbiota may play a pivotal role in preventing harmful infection, as it has been shown that some bacterial species, such as *Staphylococcus epidermis*, may prevent the adherence of others, such as *P. aeruginosa* [14]. 

Due to the devastating effects caused by this pathogen, its prevalence in healthcare settings and likelihood to present with an antibiotic-resistant strain, *P. aerguinosa* has been identified by the World Health Organization as one of the top three priority 1 pathogens requiring urgent development of new antibiotics to treat infection [8,15]. Indeed, *P. aeruginosa* is one of the most common causes of nosocomial infections, particularly in ICUs, burn units, and pediatric care [16]. This microorganism is also responsible for a large percentage of surgical site infections, urinary tract infections, and bloodstream infections [16]. Further, *P. aeruginosa*, in particular, can produce substantial virulence factors which heighten the pathogenesis of MK compared to other species [17]. These factors principally include the exoenzymes, elastases, and proteases created by this bacterium [18]. The ability of this bacterium to produce protease and gelatinase, as it invades the corneal epithelium, plays a major role in the pathogenesis of *P. aeruginosa* keratitis and makes *P. aeruginosa* keratitis a significant challenge to treat clinically [17]. These secretions are usually due to the involvement of the type III secretion system, of which the critical effector proteins have been identified as ExoU, ExoS, ExoT, and ExoY [19]. 

*Pseudomonas*, being especially nefarious, is unfortunately highly prevalent in the environment. It is often found in soil, water, and regular skin flora. In particular, it can thrive in low-oxygen environments and in moist ones, which make it likely to colonize the narrow nooks and crannies of medical equipment, as well as commonly used items such as sinks and mops [20]. Outbreaks have been linked to contaminated air conditioning systems [21], bath toys [22], hydrotherapy equipment [23], surgical equipment [24], ocular solutions [25], tap water [26], distilled water [27], and even a sample of povidone-iodine solution [28]. Regrettably, nosocomial cases in healthcare settings, where patients are most vulnerable and this opportunistic pathogen has a much greater likelihood of infecting the immunocompromised, often involve *P. aeruginosa* strains that have been selected for antibiotic resistance [15].

Finally, ocular infection following surgical interventions, or infectious endophthalmitis, is an extremely serious complication, with an incidence rate of roughly 0.1% [29]. In exceptional cases, endophthalmitis can be induced by microbial keratitis [29,30]. Endophthalmitis is caused by *Pseudomonas aeurginosa* less than 30% of the time [31], but the prognosis of *P. aeruginosa*-related endophthalmitis is especially poor, often resulting in loss of vision. In multiple studies, all strains isolated from surgical endophthalmitis cases are multidrug-resistant to cefazolin, chloramphenicol, tetracycline, aminoglycosides, and fluoroquinolones, presenting substantial challenges to saving the eye [32]. Other studies confirm a rising rate of drug resistance among *Pseudomonas* isolates [31]. However, even when organisms are sensitive to antibiotics and patients are treated promptly, over half of treated cases have resulted in either evisceration or enucleation [32].

## 3. Microorganism Adhesion to Contact Lenses

### 3.1. History of Contact Lenses

The first modern contact lenses were large blown glass scleral lenses invented in 1887 and 1888 [33,34], which corrected myopia and could only be worn for a couple of hours at a time. Plastic (polymethyl methacrylate, PMMA) scleral lenses were subsequently formed in the 1930s [35], and in 1949 the first corneal lenses were developed, which could be worn for 16 h [36]. PMMA corneal lenses became the first contact lenses to gain mass appeal by the 1960s, but these lenses were not oxygen permeable and carried a range of potential comfort and clinical side effects. Rigid gas permeable contact lenses became available in the late 1970s and quickly became the industry standard for rigid lenses [37]. In 1998 the first silicone hydrogel contact lenses were released by Ciba Vision. These soft lenses combine high oxygen permeability with comfort and high clinical performance and have since become the most common type of contact lens dispensed [38]. 

### 3.2. Material Properties 

While environmental and microbially intrinsic factors undeniably contribute to device adhesion by microorganisms, these areas are usually beyond the direct control of a device manufacturer. The polymers used to create medical devices are, however, subject to design choice. In this section, we review the material properties that are widely regarded as affecting microbial adhesion. 

Microbial adhesion can be conceptualized as a thermodynamic system, composed of an organism, a surrounding liquid, and a substrate, progressing to a lower free energy state. Part of the total free energy of this system is attributed to the free energies found within the surface-to-surface interaction of the system components: liquid-to-organism, liquid-to-substrate, and organism-to-substrate. These interfacial free energies can be used qualitatively to predict bacterial adhesion to a substrate [39], however, quantitative predictions are often limited by measurement difficulties. Still, material properties that contribute to the interaction energy can often be measured accurately. 

A conventional measure of the surface stiffness of a material is the Young’s or Elastic modulus with units of Pascals (Pa). The Young’s modulus of a polymer is commonly determined through the use of atomic force microscopy to measure the force required to indent a surface to a given depth. The Young’s modulus can be approximated for the material using a Hertzian model of displacement and force. The surfaces of soft hydrogel contact lenses typically have Young’s moduli of between 0.2 and 2.0 MPa [40]. 

Song et al. investigated the effects of stiffness of polydimethylsiloxane (PDMS) polymer samples on bacterial adhesion [41]. The authors created PDMS samples with Young’s moduli ranging from 2.6 MPa to 0.1 MPa increasing the ratio of base elastomer to curing agent from 5:1 to 40:1. The authors performed a kinetics study of the extent of bacterial colonization of the differing PDMS samples with time points from 2 h to 72 h. They observed that at early time points, stiffer PDMS samples (2.6 MPa) consistently colonized with up to 2-log fewer *E. coli* per square centimeter than softer samples (0.1 MPa). Similar results were seen for *P. aeruginosa*. This trend was diminished by prolonging the contact time or by using increasingly dense initial inoculum cultures. Interestingly, the authors also observed phenotypic changes to bacteria adhered to soft surfaces, including longer average cell length and decreased survivability when challenged with antibiotics.

In a similar study by Straub et al., investigators created a PDMS series with Young’s moduli ranging from 4.52 MPa to 0.06 MPa [42]. They observed that for *E. coli* and *P. aeruginosa*, bacterial adhesion was inversely proportional to stiffness over the range of 0.06 to 3.91 MPa. Interestingly, this trend was not seen for the gram-positive organism *S. aureus*, which maintained similar binding levels to all samples regardless of stiffness. A direct causal interpretation of these findings is complicated by other factors that correlate with stiffness. The authors observed that, while all samples could be considered hydrophobic, the water contact angles for the differing PDMS samples were inversely proportional to the Young’s moduli. In a follow-up publication, the group performed experiments to observe detachment of *E. coli* from PDMS substrates of different stiffnesses while under flowing conditions. The authors observed *E. coli* bound to softer surfaces were largely immobilized, whereas bacteria adhered to stiffer surfaces were easily moved or dislodged [43]. 

Kolewe et al. published an analysis of the stiffness–colonization relationship for polyethylene glycol (PEG)-based hydrogels for *E. coli* and *S. aureus* [44]. In contrast to the observations of Song et al. [41]. and Straub et al. [42], the authors found that for PEG hydrogels with Young’s moduli from approximately 0.02 MPa to 3 MPa, *E. coli* and *S. aureus* colonized stiffer materials to a greater extent than softer materials. This contradiction may indicate the importance of material properties other than stiffness or may even cast doubt upon the importance of stiffness in relation to bacterial adhesion. However, it is worth noting that Kolewe et al. performed their assays using 24 h contact times, in contrast to the shorter durations (as little as 2 h) used by Song et al. and Straub et al.. In light of this technical difference, one must consider that results derived from long incubation periods may reflect the effects of multiple biological mechanisms that contribute to colonization of a substrate. These kinetically slower mechanisms, such as biofilm formation or macromolecular deposition, represent potential confounds that prevent definitive claims about bacterial adhesion to a naive surface. Indeed, in the kinetics experiments of Song et al. [41], stiffer PDMS samples were found to lose their bacterial adhesion advantage over softer materials at time points beyond 24 h. Ultimately, bacteria respond actively to material stiffness [45]. 

Finally, there are some highlighted overall findings which can be summarized from the body of work so far completed regarding material properties affecting contact lens microbial adhesion, some of which will be discussed in more detail in other sections. For instance, contradictory studies have been published regarding which inherent properties of lenses promote or prevent bacterial adhesion. Some examinations indicate hydrophobic lenses adhere greater levels of bacteria than hydrophilic ones [46,47,48], while others indicate the opposite [49]. There have been widely varied reports regarding water content of a contact lens and adhesion and which type of lens is investigated, and the effects of water content may be over shadowed by polymer composition [14]. In general, microorganisms bind at higher rates when surface roughness is increased, and worn lenses often display increased surface roughness compared to unworn lenses [14]. The surface roughness of worn lenses is due to protein deposition, usually albumin, globulin, and lysozyme in tears, and lipid deposition, stemming from the meibomian glands of the lids [14]. Thus, lenses may accumulate adsorbed protein and lens surface deposits, and bacteria has been shown to adhere to these areas of lens deposits [14]. However, once lipids are adsorbed to lenses, they appear to have no effect on bacterial adhesion. Additionally, bacterial adhesion is reduced if lenses are coated with secretory phospholipase A2 due to its ability to cleave fatty acids from phospholipids, thereby damaging bacterial membranes [14]. Bacterial species have been shown to adhere differently to ionic or non-ionic lenses. For instance, *P. aeruginosa* adheres to lenses composed of non-ionic polymers as compared to those made of ionic polymers, while *S. aureus* adherence was higher to an ionic hydrogel as compared to a non-ionic hydrogel [14]. However, among all else, the results regarding lens water content are remarkably consistent: bacterial adhesion increases inversely with lens water content [48,49,50,51,52,53].

### 3.3. Adherence Mechanisms

**Figure 2:**
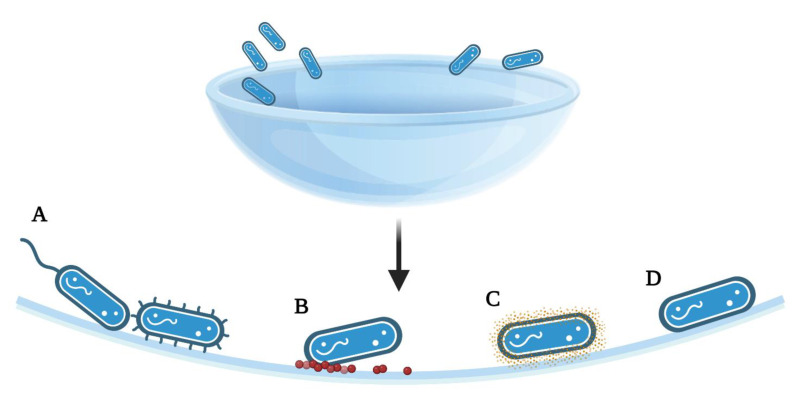
Summary of the adhesion strategies, particularly to achieve a smaller separation from the surface than allowed by the initial energy minimum. (**A**) Attachment of bacteria via flagella or pili, (**B**) Surface deposits of proteins or lipids following wear, allowing for bacterial attachment, (**C**) Production of adhesive proteins, polysaccharides, or extracellular polymeric substances exposed on the cell surface (**D**) Polar adhesion; [2,14]. The figure is created using BioRender.com. (accessed on 18 March 2022).

Microorganism attachment to contact lenses is mediated by a range of potential forces, properties, or active strategies (Figure 2). Classical DLVO theory (Derjaguin, Landau, Verway, and Overbeek) assumes that bacteria and the substrates are smooth and of uniform chemistry, thereby disregarding surface roughness, charge, chemical non-uniformity, or hydrophobic or solvation forces [54]. This theory allows investigators to also determine the attachment forces involved in cell adhesion, without appendages or other mechanistic strategies. Multiple innate forces act on microorganisms as they approach a contact lens: long-range Van der Waals forces will initially attract an organism, but the short-range repulsive electrostatic forces produced by both surfaces being negatively charged may repel it [2]. Acid–base and hydrophobic interactions may further attach or repel the microorganism from the surface depending on the relationship, but as noted, this relationship has been shown to both enhance and discourage adhesion [2]. 

As discussed, microorganisms can demonstrate improved attachment to rough surfaces, whether the surface roughness is from the matrices itself or accumulated proteins, lipids, or even other microorganisms. However, more active processes are found in the form of proteins expressed on the cell surface of microbes which mediate attachment to biotic and abiotic surfaces. Pilins, which are fimbrial adhesins, are a class of proteins represented across phylogenetic domains that form filamentous structures anchored to the cell surface. Pilins can enable many cellular functions, including pathogenesis, by facilitating microbial adhesion to a variety of surfaces, motility, cell-to-cell aggregation and biofilm formation, and genetic exchange. Pili of gram-positive bacteria may use covalent links between fimbrial subunits to produce the pilus fiber [55]. Specifically, pili enable the nonspecific interactions that govern the attachment to and motility on inert surfaces, and pilicide activity has been noted to inhibit bacterial adherence in some strains [55]. *P. aeruginosa* express a type IV pilus (T4P) that is particularly relevant to ocular pathogenesis. The T4P of *P. aeruginosa* mediates twitching motility, a gliding movement of the organism along a material surface that does not depend upon the flagellar motility system. Twitching motility has been shown to enable *P. aeruginosa* to move against fluid flow on a PDMS surface. [56] In addition to the contribution of T4P to abiotic surface interactions, invasion of corneal epithelial cells by *P. aeruginosa* is facilitated by T4P [57]. Interestingly, both the adhesive properties and twitching motility functions of T4P are required for corneal infectivity, as demonstrated with a murine model. [58]

Flagella, which are motile and highly hydrophobic, have been found in some strains and species to increase adherence, such as in *E. coli*, and in others flagella have been found to reduce adhesion, such as in *Caulobacter crescentus* [2]. Flagella have been shown to initiate cell signaling once attached, which promotes swarming [2]. However, flagella possess a variety of sensing mechanisms under both adhered and non-adhered states, which further contribute to the ability of flagellated bacteria to accumulate [2]. In general, flagella and pili both facilitate adhesion to inert surfaces, but also may transmit signals that allow bacteria to respond to and potentially accelerate adhesion. T4P and flagella may be used to explore a surface by moving or swarming over it before irreversible attachment is achieved [2]. Some cells may detach, maintaining multigenerational memory mediated by T4P activity that can prepare them for a stronger adhesion when a new surface is encountered [2]. Thus, following irreversible adhesion, bacteria can then stick together by producing an exopolymeric matrix and their appendages, by synthesizing adhesins [2].

In addition to the adhesion mediated by appendages, some investigations have indicated that envelope stress can be exploited by the cell as a signal for surface attachment [2]. Indeed, in *P. aeruginosa* specifically, surface-induced stress could be a trigger for biofilm formation [2]. Flow and sheer stress can also cause the production of c-di-GMP and subsequent biofilm initiation in *P. aeruginosa* that were attached via T4P. However, the mechanism for sensing the shear rate in bacteria remains unknown [2]. Lastly, in addition to the signaling pathways and proteins that are required for adhesion, sessile cells in a biofilm also undergo alterations to downregulate motility [2].

Some key proteins have been shown to be critical in adhesion processes. For instance, LptF is an outer membrane protein which may be required *P. aeruginosa* to survive in harsh environments [59] and FliD is a flagellar cap protein which facilitates the adhesion process [60]. However, a host of critical proteins have been identified in MK-causing organisms which are required or beneficial for adhesins and adhesion. These include YeeJ (*E. coli*), FimA (*E. Coli*, *S. marcescans)*, CdrA (*P. aeruginosa*), among many others [61]. 

Finally, microorganisms can facilitate adhesion via the production of an exopolymeric matrix, such as polysaccharide colanic acid or poly-β-1,6-N-acetyl-D-glucosamine in *E. coli*, or alginate, Psl, and Pel in *P. aeruginosa* [2].

### 3.4. Pseudomonas Adherence to Contact Lenses

*Pseudomonas* species are the most commonly isolated organism from contact lens-related MK [14], and *Pseudomonas aeruginosa* adheres in the highest numbers of any organism tested so far to both hydrogel and silicone hydrogel lenses in vitro [14]. Williams et al. used variable viable high cell counts on contact lenses to try to understand if more bacteria led to more adhesion and tested five different *P. aeruginosa* strains to investigate [62]. Interestingly, it appears that only about 10% of the cells in any *Pseudomonas* population are responsible for most of the adhesion, but this may depend on the strain being investigated and in what growth phase the examination takes place [14,62]. *Pseudomonas* stands apart from other species due to its large genome [57] and highly dynamic proteome, [63] giving it more opportunities to adapt to environmental challenges or overcome antibiotic contests. In regard to adhesion, it is able to adhere to the surface of the contact lens or the cornea via flagellum (fliC), pili (pilA), or body [64]. George et al. determined, through a series of time course studies of adhesion through ATP or radiolabel analyses (using a 37% water-content hydrogel lens across experiments), that *Pseudomonas* is additionally able to adhere to a lens in as little as five minutes, while other commonly isolated species can take two or three hours to adhere [14,65]. This ability to adhere quickly appears to be similar across many lens types, and this quickness to adhesion may be one of the key reasons this organism is responsible for such a large percentage of MK cases. 

As has been shown in similar examinations, any magnitude of surface deposits–such as those protein and lipid depositions that appear during the normal course of contact lens wear–greatly increases the adhesion of *P. aeruginosa* to contact lenses [66]. This was determined by Butrus et al. by using both high and low inoculums of *P. aeruginosa* and examined with a wide variety of lens treatments including neuraminidase, mannosidase, glucosidase, and alkylcarboxylic acid for up to 48 h, all using extended-wear contact lenses [66]. Likewise, Simmons et al. determined by using hydrogel contact lenses from four of the FDA groups (those available when the study was done) and 12 h suspensions of 1 × 10^7^ CFU/mL of *P. aeruginosa* (ATCC 27853) that the formation of *Pseudomonas* biofilm formation on a lens enhances the ability for other pathogenic microorganisms to bind to the lens as well [67]. It is also evident that due to *Pseudomonas*’ abundance of genes, that contact lens disinfection methods select for the most cytotoxic bacteria, as the cytotoxic isolates are also the most resistant to disinfection methods. [68] Indeed, the persistent isolates collected from contact lenses and contact lens solution from an eye hospital were also identified as multi-drug resistant, biofilm forming strains [69]. Importantly, *Pseudomonas*, and other contact lens-related microorganisms, have been shown to be much harder to disinfect once they become biofilms, as opposed to planktonic cells [70]. Finally, extended-wear contact lenses, which are meant to be worn continuously for up to four weeks (as opposed to contacts which are removed and disinfected daily, or daily lenses which are disposed of after 12–16 h of wear) have been shown to be especially susceptible to *Pseudomonas* contamination [71]. While these extended-wear lenses are less likely to be contaminated via handling in tap water or used contact lens care solution, they are highly susceptible to contamination via the patient wearing them while swimming or in the shower, or otherwise touching their eye during wear [71]. These lenses have been shown to have the greatest amount of protein and lipid deposition of all lens types, and it has been found that extended contact lens wear significantly increases the bacterial adherence to corneal epithelial cells directly [72].

## 4. Methods to Evaluate Contact Lens *Pseudomonas* Adhesion

### 4.1. In Vitro Analyses

The industry standard for determining microbial persistence after contact lenses have been challenged with microorganisms and disinfected by contact lens care solutions is governed by the International Standards Organization Standard 18259 protocol and 14729 protocol [73,74]. However, this protocol does not necessarily provide methodological guidance on quantifying microbial adhesion to contact lenses, so investigators have developed a variety of methods in order to analyze adhesive properties and quantities of microorganisms on contact lenses. 

Qualitative analysis can be achieved by imaging microbes attached to lenses using brightfield or fluorescent microscopy, which may require finding ways to make microorganisms easier to visualize, either via transgenic modification or fluorescent stain [75]. Morphology and adhesion have also been analyzed via scanning electron microscopy and transmission electron microscopy [70,76]. Contact lenses themselves have also been noted to have ultraviolet autofluorescence, which could affect microscopic evaluation [77]. Most quantitative methods revolve around removing the microorganisms from the contact lens after the adhering period in order to then quantify the removed population in solution or on a microscope slide. Microorganisms are removed from lenses via a variety of methods including sonication [70,75], vortexing [78], and vortexing with glass beads [79]. Following the removal of organisms from the lens and their resuspension in solution, microorganisms are usually serially diluted, plated on the appropriate agar, and the colony forming units are enumerated following incubation [70,75,78,79]. 

Some analysis methods rely on active reactions, such as luminescence or radiation in order to quantify viable adhered cells. The measurement of adenosine trisphosphate (ATP) of microorganisms is often used to quantify adherence by measuring luminescence via ATP assay [80]. ATP assay can be performed by transferring lenses, with microorganisms attached, to a luminometry plate where ATP is extracted from viable cells. Upon retrieving the sample from the plate, a luciferase-luciferin reagent is added, and light output can be measured [65]. From this, a calibration curve relating light output to viable cell counts can be generated by comparing light output to a serial dilution cell count curve from the original inoculum [65]. Similarly, radio-label cell analysis is possible by adding lenses to scintillation vials with suspensions of radio-labeled microorganism cell suspensions [65]. After an incubation period, lenses with adhered microorganisms can then be rinsed off the lens and microorganism samples can be put into a scintillation cocktail. Scintillation counts are normalized for background radiation and converted to cell numbers via a calibration curve which relates disintegrations per minute to viable cell counts established from inoculum controls [65]. However, while direct correlation data between all of the above methods is limited, it has been shown that differing bacterial recovery and quantification methods can produce highly different results [81]. 

### 4.2. Animal Studies

A variety of well-documented animal models have been used by investigators to examine microbial adherence to contact lenses. Of these, the most often cited models include rabbits [82,83,84], mice [85,86], rats [87,88], and guinea pigs [82,89]. The New Zealand White rabbit is one of the most commonly used animals overall [89]. These models are most often used to assess contact lens-induced acute red eye (CLARE) and contact lens-induced peripheral ulcers (CLPUs). Some animals are also genetically modified in order to further examine host factors involved in disease, such as mice deficient in cytokines and other immune factors [89]. In regard to examining microbial adherence to contact lenses specifically, a common protocol involves anesthetizing the animal or providing local ocular anesthetic, then scratching the cornea in order to imitate the corneal scratches which allow for corneal infection in patients, applying an appropriately sized and fitted contact lens (with or without known microbial adherence occurring on the lens), and potentially suturing the eyelid closed to keep the lens next to the cornea for an experimental period (usually between 12 and 48 h [89], but can go up to many days [90]). The contact lens is then removed, and the animal is monitored for signs of infection, or the eye is removed for examination. In vivo, fluorescein stain is the standard examination tool used to visualize corneal or stromal infiltrates and the degree of corneal erosion present, and veterinary slit lamp examinations can also permit three-dimensional visualization and measurement of the finer anatomy of the eye.

## 5. Adhesion of other Microorganisms to Contact Lenses

Five significant microorganisms are required testing for manufacturers of contact lens care solutions when demonstrating antimicrobial efficacy: *Pseudomonas aeruginosa*, *Staphylococcus aureus*, *Serratia marcescens*, *Candida albicans*, and *Fusarium solani* [91]. While not yet required for testing, *Acanthamoeba* is now also of significant importance to the ophthalmological community due to the seriousness of potential *Acanthamoeba* infections [92].

*S. marcescens* has been determined as an important microorganism in regard to contact lenses due to the prevalence of its isolation from MK cases [14]. Notably, *S. marcescens* has been found to adhere increasingly to contact lenses as the lens water content decreases. [93] Similar to what has been found in *Pseudomonas*, *Serratia* is able to produce protease and gelatinase which may play a role in the pathogenesis of contact lens-related *Serratia* keratitis [94]. The prevalence of *Pseudomonas aeruginosa* as the leading pathogen associated with MK [14] is followed by *Staphylococcus aureus*. *Staphylococcus* adhesion to lenses varies between strains and lens types, but it has been found that slime-producing strains have a higher rate of adhesion than non-slime strains [95]. Similarly, *Staphylococcus* strains which form biofilms have been shown to adhere to lenses at higher rates than those which do not form biofilms [71].

Fungal and amoebal infections are particularly dangerous forms of keratitis due to the limited treatment options. Adhesive mannoproteins in fungus and *Acanthamobea* are essential to the microorganisms’ ability to bind to the corneal epithelium [11]. These proteins recognize D-mannose or mannose glycoproteins of the epithelium and damage to the cornea results in the upregulation of these mannose glycoproteins, potentially contributing to a positive pathogenic feedback loop once the adhesion process begins [11,12]. To note, the upregulation of mannosylated glycoproteins are often a response to corneal abrasions, and a response to contact lenses, and are the pathophysiological link between ocular injury and MK [12]. Microorganisms may also use binding sites such as laminin, fibronectin, and collagen [11]. Several common tear proteins such as albumin, lactoferrin, and lysozyme, as well as fibronectin, have been found to increase the adherence of *Candida* to contact lenses [96]. As with other microorganisms, studies have determined a strong correlation between *Candida* adherence and water content in lenses [96]. Both *Fusarium* and *Candida* have been shown to form biofilms on many types of lenses, and these biofilms have reduced susceptibility to contact lens care solutions [97]. *Fusarium* is also unique for its formation of penetration pegs, which are hyphae of the fungi that traverse into the matrix of a contact lens [98]. While worn lenses have repeatedly been shown to be more likely to adhere more microorganisms in general, *Fusarium* has demonstrated increased rates of penetration pegs in worn lenses, but not necessarily increased adhesion [98].

*Acanthamoeba* keratitis is a rare but extremely serious infection. As an amoeba, it is profoundly difficult to treat once infection has begun, with as many as 15% of patients losing vision in the affected eye [99]. As with other pathogens, studies have confirmed that contact lenses with rougher surfaces have a higher rate of *Acanthamoeba* adhesion than lenses with smoother surfaces. [100] Similarly, multiple studies have indicated that the formation of biofilms by bacteria such as *Pseudomonas* enhance the adhesion of *Acanthamoeba* to contact lenses [67]. 

## 6. Antimicrobial Contact Lenses

As previously noted throughout this review, the smoother the surface, the less likely a microorganism is able to attach to it. However, antimicrobial additives or alterations to materials to prevent microbial growth has been an expanding field of research for decades, and recent investigations have indicated that adding antimicrobial properties to contact lenses may help prevent microbial keratitis by limiting microbial binding. Among these strategies, silver nanoparticles have been extensively studied for their antimicrobial properties, and there are silver-impregnated contact lens cases commercially available [90]. However, while contact lenses with silver nanoparticles have been investigated, they are currently not feasible due to the cost of the material but also due to the toxic effect of their positive charge [90]. Combinations of tannic acid-modified silver nanoparticles with contact lens solutions have recently been shown to be effective against the adherence of *Acanthamoeba* when combined with contact lens care solutions [101]. Following up on the silver studies, zinc and copper are likewise being investigated for their potential to be added to contact lenses to provide innate antimicrobial properties. These metals have been demonstrated to be promising, but in vivo examinations are lacking, and these nanoparticles require further attention [90]. 

Overall, the majority of the strategies investigated do show promising efficacy in either antimicrobial properties or in preventing microbial adhesion, but common drawbacks to any method include cost or cornea cell toxicity [90]. Other significant barriers to success in reducing the prevalence of MK include the extremely high rates (up to 99%) of contact lens wearers who do not follow the necessary directions to keep their lenses pathogen-free. These dangerous habits include not performing the rub and rinse step daily, reusing non-reusable lenses, rinsing lenses in tap water, swimming or showering while wearing lenses, not washing hands before applying contact lenses or touching the eye, and using topped-off contact lens care solution (i.e., not completely replacing the solution in the lens case nightly, but instead only adding a small portion to replace what was lost overnight, which leads to biocide uptake and reduces the effectiveness of the solution) [102]. It is possible that the most successful antimicrobial additive strategy will include a combination of additives or additives used in conjunction with other disinfection tools. However, it should be noted that studies show that residual cellular debris on contact lens surfaces can contribute to new colonization and can effectively neutralize additive lens-based antimicrobial strategies [75], which encourages the idea that the material properties of contact lenses that naturally repel microorganism adhesion should be a goal of any manufacturer. 

## 7. Conclusions

In conclusion, microbial keratitis remains a significant risk for contact lens wearers, and the common microorganisms causing this devastating disease are becoming harder to neutralize once infection sets in. Among these, *Pseudomonas aeruginosa* stands out as a model organism to be studied due to its overwhelming prevalence as the causative organism of MK cases, and due to the severity of the disease it causes. Thus, *P. aeruginosa* should be used as a primary microbial investigation tool to evaluate adherence to contact lenses and develop improved products available to patients. A multitude of strategies to prevent the binding of this pathogen are available but contact lenses that are innately resistant to the binding of this microbe are needed to improve patient safety.
